# Health Inequalities in German Higher Education: A Cross-Sectional Study Reveals Poorer Health in First-Generation University Students and University Students with Lower Subjective Social Status

**DOI:** 10.3390/ejihpe16010011

**Published:** 2026-01-05

**Authors:** Corinna A. Södel, Marga Motzkau, Marcel Wilfert, Raphael M. Herr, Katharina Diehl

**Affiliations:** 1 Department of Medical Informatics, Biometry and Epidemiology, Friedrich-Alexander-Universität Erlangen-Nürnberg (FAU), 91054 Erlangen, Germany; 2 Bavarian Cancer Research Center (BZKF), 91054 Erlangen, Germany; 3 Comprehensive Cancer Center Erlangen—European Metropolitan Area of Nürnberg (CCC ER-EMN), 91054 Erlangen, Germany

**Keywords:** health inequalities, subjective social status, first-generation students, socioeconomic status, university, self-rated health, well-being, stress, depression, burnout

## Abstract

University students worldwide experience considerable health challenges. We examined health inequalities in a nationwide, gender-balanced sample of 1105 German students, considering negative (stress, depression, burnout) and positive health outcomes (self-rated health [SRH], well-being) alongside vertical (subjective social status [SSS], parental academic background) and horizontal (gender) determinants. Analyses used bivariate statistics, multivariate regressions, and interaction terms. Higher SSS was associated with better SRH (β = 0.322) and well-being (β = 0.355), and lower stress (β = −0.154), depression (β = −0.127), and burnout (β = −0.219). First-generation students reported highly significant poorer SRH and well-being than students with one (β = 0.114; β = 0.112) or two academic parents (β = 0.162; β = 0.192). Students with two academic parents showed lower stress (β = −0.087, *p* = 0.007) and burnout (β = −0.099, *p* = 0.002). Interactions suggest a protective effect of higher SSS on depression (β = −0.219, *p* = 0.026) and burnout (β = −0.264, *p* = 0.006), more pronounced among male students, who additionally benefited more from an academic household regarding SRH (β = 0.100, *p* = 0.044). These findings underscore intersectional and multifaceted inequalities among German students and the need for interventions.

## 1. Introduction

Mental health problems are widespread among university students, with a lifetime prevalence of mental disorders ranging from one-third (Auerbach et al., 2018) to two-thirds (Mason et al., 2025) of the student population. Recent studies have documented a growing burden, with high levels of stress (Brett et al., 2023), depression (Backhaus et al., 2020), and burnout (Liu et al., 2023) among university students. However, individual factors such as gender contribute to differences in students’ health (Anis-Farahwahida et al., 2024).

For instance, female students consistently showed higher levels of stress (Brett et al., 2023; Gellisch et al., 2024) and a higher prevalence of depression (De Groot et al., 2024; Worthen et al., 2023). Furthermore, male students reported higher levels of self-rated health (Kim, 2022) and well-being (Brett et al., 2023). Findings regarding burnout have been inconsistent, with a high prevalence found in both male (Liu et al., 2023) and female students (Fiorilli et al., 2022).

Beyond gender as an individual-level horizontal factor, socioeconomic characteristics as vertical determinants constitute a key dimension of health inequality (Lampert et al., 2013). Horizontal health inequality refers to differences in morbidity and mortality that can be attributed to demographic characteristics such as age or gender (Hradil, 2009), while vertical health inequality describes systematic health differences between socioeconomic classes (Richter & Hurrelmann, 2006). However, traditional indicators such as income, education, and occupation are often less meaningful for university students and difficult to measure in this target group (Diehl et al., 2021). An established measurement tool for individual socioeconomic circumstances is the subjective social status (SSS), which reflects how individuals rank themselves relative to others (Adler & Stewart, 2007; Diehl et al., 2017). Research has shown that SSS is a better predictor of mental health and well-being than objective social status (Haught et al., 2015; Hoebel et al., 2017), which emphasizes its relevance for understanding health inequalities in the student population.

While SSS captures one dimension of vertical inequality by focusing on perceived individual status, first-generation status at the university represents another important aspect of vertical inequality (Diehl et al., 2022). Although definitions vary, first-generation university students (so-called first-gens) are typically defined as students whose parents do not hold a university degree (Maurici-Pollock et al., 2025). First-gens often have less educational, cultural, and social capital (Barsegyan & Maas, 2024) and tend to feel a lower sense of belonging at the university (Gopalan & Brady, 2020; Lawrie & Kim, 2024). These aspects, in turn, are associated with lower academic performance, higher dropout rates, and further challenges (Barsegyan & Maas, 2024; Drake, 2024; Gopalan & Brady, 2020; Lawrie & Kim, 2024; Rockwell & Kimel, 2025).

It has been shown that SSS and first-gen status are associated with negative health outcomes among students (Mac-Ginty et al., 2024). For example, SSS has been related to increased levels of depression (Backhaus et al., 2020; Diehl et al., 2021; Niu et al., 2023; Pössel et al., 2022) and stress (Gellisch et al., 2024; Huynh et al., 2023; Sendatzki & Rathmann, 2022). Similarly, first-gens reported symptoms of stress (Williams-York et al., 2024), and depression (Amirkhan et al., 2023; Worthen et al., 2023) more often, although two studies found no significant differences (Huynh et al., 2023; Noel et al., 2023) compared to their peers. A systematic review of the literature highlighted that a lower family social status was associated with burnout in students (Zahra & Hidayat, 2024). Furthermore, initial studies indicate that first-gens were more likely to experience burnout (Liu et al., 2023; Williams-York et al., 2024).

These associations are not limited to mental health burdens; they also extend to more general measures of health and well-being. For instance, SSS was positively associated with self-rated health (Diehl et al., 2021; Sendatzki & Rathmann, 2022), whereas first-gen status was not significantly associated with self-rated health (Deindl et al., 2023). However, parental occupation status was positively associated with students’ self-rated health, suggesting that socioeconomic and cultural capital may be important for first-gens’ health (Deindl et al., 2023). Students with higher SSS tended to describe higher levels of well-being (Niu et al., 2023), whereas first-gens reported lower levels (Qin et al., 2024).

Existing research on students’ health has primarily focused on negative outcomes such as stress and depression, while salutogenic indicators like self-rated health and well-being have received less attention (Hernández-Torrano et al., 2020). Moreover, many studies focused on single universities (Huynh et al., 2023; Kim, 2022; Sendatzki & Rathmann, 2022) or specific groups, such as health sciences or medical students (Gellisch et al., 2024; Williams-York et al., 2024; Worthen et al., 2023), and often lack gender balance (De Groot et al., 2024; Diehl et al., 2022; Gellisch et al., 2024; Huynh et al., 2023; Pössel et al., 2022; Sendatzki & Rathmann, 2022; Worthen et al., 2023). Based on previous research, we developed the following research question: How are SSS and first-gen status associated with positive and negative health outcomes among German university students, and to what extent does gender moderate these associations?

**Figure 1:**
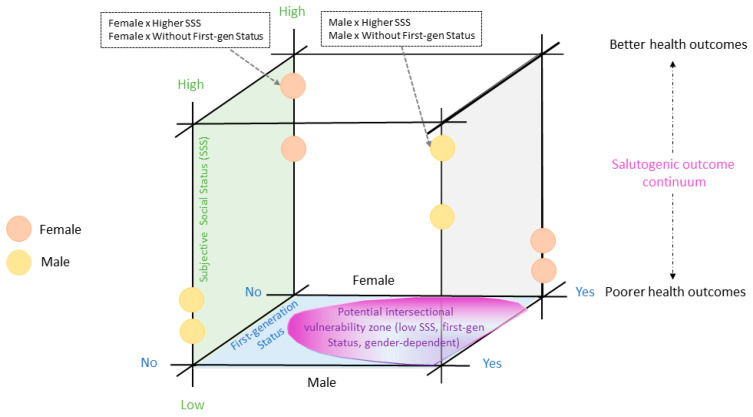
Conceptual framework of this study.

Figure 1 illustrates the conceptual framework of this study. Gender is displayed on the horizontal axis, SSS on the vertical axis, and first-gen status on the base of the figure. The potential intersectional vulnerability zone (low SSS, first-gen status, gender-dependent) indicates contexts in which interaction of these factors may be associated with poorer health outcomes. Positions outside this zone may be associated with better health outcomes, conceptualized along a salutogenic outcome continuum.

Building on the research question and the conceptual framework of this study, the aims of our research were to achieve the following:

1. Gain a broader perspective in line with the salutogenic framework by considering not only the negative health outcomes (stress, depression, burnout), but also the positive ones (self-rated health, well-being).
2. Contribute to a more nuanced understanding of students’ health by addressing a subjective dimension (SSS) and an objective dimension (first-gen status) of vertical health inequalities.
3. Assess the combined relationships of gender and vertical dimensions to recognize the multifaceted interactions of social determinants in relation to university students’ health. In doing so, the study also advances ongoing efforts to quantitively ground initial indications of intersectional perspectives (LeBouef & Dworkin, 2021).

By addressing these objectives, our study provides a basis for specific recommendations regarding prevention and support strategies for socially diverse student groups.

## 2. Materials and Methods

### 2.1. Participants

Participants for this study were randomly selected from an online panel of a social and market research institute (https://bilendi.de/, accessed on 23 September 2025). High-quality standards were maintained throughout the survey. For example, cases with unusually short response times (“speeders”) or uniform response patterns (“straight liners”) were excluded. In addition, the Working Group of German Market and Social Research Institutes (ADM) ensured that the panel consisted only of natural persons and not bots. Moreover, it was guaranteed that each participant could complete it only once. The quota sampling ensured that the target group was balanced by gender (50% female students and 50% male students) and by university type (70% university students and 30% university of applied sciences students), representative of the German student population. The inclusion criteria were current enrollment at a university and sufficient knowledge of German. A total of 1105 German students gave their consent and participated in the study (Herr et al., 2022). They were compensated for their participation via the social and market research institute’s bonus system. The Ethics Committee II of the University of Heidelberg (2019-1123N, 31.01.2019/19.10.2020) approved the study.

### 2.2. Measurements

**Subjective Social Status (SSS).** On a student-adapted 10-point Mac Arthur scale (Adler & Stewart, 2007; Diehl et al., 2017), participants rated their SSS. Students were asked to place themselves on an imaginary ladder relative to their peers. The top rung represented students with the highest income and highest levels of education, and the bottom rung represented those with the lowest income and lowest levels of education (Diehl et al., 2017).

**Parental academic background.** To determine the parental academic background, participants answered whether their parents held a university degree. Those who responded “No, neither parent” were classified as first-gens. Participants who indicated that one parent (“Yes, one parent”) or both parents (“Yes, both parents”) hold a university degree were considered to have a parental academic background.

**Self-rated health.** Participants assessed their health status using the single validated item of the Self-Rated Health (SRH) scale, on a five-point scale ranging from 1 (“very poor”) to 5 (“very good”). The SRH item has been shown to predict various future health outcomes, including mortality and psychosocial health (DeSalvo et al., 2006).

**Well-being.** The WHO-5 Well-Being Index (WHO-5) is a psychometric instrument, which is designed to assess mental well-being (WHO, 2024). The scale demonstrated a high internal consistency in our sample (Cronbach’s α = 0.900). Participants indicated how often they had experienced positive well-being over the past two weeks by responding to five items on a six-point Likert scale ranging from 0 to 5. Lower scores have been shown to indicate the need for further assessment for potential mental health conditions (Sischka et al., 2020).

**Stress.** The German extra-short version of the Stress Overload Scale (Amirkhan, 2012), the SOS-X-G (Haehner et al., 2023), is a validated self-report measurement. Cronbach’s α was 0.891 in the current sample. Mean scores were calculated from four items rated on a four-point Likert-scale ranging from 1 (“not at all”) to 5 (“completely”), with higher scores indicating greater perceived stress.

**Depression.** Depression was measured using the Patient Health Questionnaire 8 (PHQ-8). PHQ-8 is a validated instrument with strong psychometric properties for assessing depression in the general population (Kroenke et al., 2009) and in students (Alpizar et al., 2018). The scale showed good internal consistency (α = 0.882). The mean score was computed from eight items rated on a four-point Likert scale (1–4), with higher scores indicating greater severity of depressive symptoms.

**Burnout.** The Maslach Burnout Inventory-Student Survey (MBI-SS) in the German version was used to assess burnout in the target population (Gumz et al., 2013). The three dimensions of emotional exhaustion (α = 0.772), cynicism (α = 0.910), and academic efficacy (α = 0.869) were assessed using a total of 15 items (Maslach et al., 1997). Responses were given on a seven-point Likert-scale ranging from 1 (“never”) to 7 (“always”). Items for academic efficacy were reverse-coded, whereas those for emotional exhaustion and cynicism were not recoded, to ensure that higher mean scores across all three dimensions reflect greater levels of burnout.

**Sociodemographic, additional socioeconomic and study-related characteristics.** Gender was assessed using the question, “Which gender do you feel you belong to?”. As this phrasing reflects a psychological understanding of gender identity, the term gender is used throughout the study rather than sex. Migration background was categorized into two groups (yes; no), using three questions concerning country of birth, parental origin, and mother tongue (Schenk et al., 2006). Age (in years) was also considered as a sociodemographic variable. To capture the additional socioeconomic characteristics, participants were asked to indicate their primary source of income (support from parents, relatives, partner; funding of Federal Law on Support in Education; own employment; scholarship, loans and others) and their living situation (alone; with partner; in shared apartment; student dormitory; with parents or relatives). Study-related characteristics included the type of university (university; university of applied science), area of studies (humanities; law, economics, and social sciences; mathematics, natural sciences; medicine, health sciences; engineering; art, art science; sports; others), and current semester (1–2; 3–6; >6).

### 2.3. Statistical Analyses

First, descriptive statistics (means, standard deviations, proportions) were computed to provide an overview of the study sample.

Second, we conducted *t*-tests, ANOVAs, and Pearson’s correlations to examine associations between SSS and students’ characteristics. Additional bivariate analyses were performed using Chi^2^-tests including post-hoc tests and an ANOVA to assess the associations between the categorical variable of parental academic background and students’ sociodemographic, additional socioeconomic, and study-related characteristics.

Third, linear regression models were used to examine the relationship between SSS and health outcomes in the total sample, as well as separately for female and male students. Four models were estimated for each outcome:

- I: unadjusted model (without covariates)
- II: sociodemographic model (adjusted for age, migration background, gender)
- III: socioeconomic model (adjusted for primary source of income, living situation)
- IV: study-related model (adjusted for type of university, area of studies, semester)

Fourth, analog to the previous step, four linear regression models (I–IV) were also used to assess the relationship between parental academic background and health outcomes. Given that the parental academic background was a categorical variable, first-gens served as the reference group in these analyses.

Fifth, additional analyses aimed to determine whether gender functioned as a moderating factor in the potential associations between health outcomes and SSS and the parental academic background. Therefore, interaction terms were included. The preconditions for regression analyses, such as normal distribution of variables and non-multicollinearity of covariates, were carefully checked.

## 3. Results

### 3.1. Participant Characteristics

The study comprised 50.1% (*n* = 554) female students and 49.9% (*n* = 551) male students, as well as 67.2% (*n* = 743) university students and 32.8% (*n* = 362) students from universities of applied sciences. The study sample (Table 1) showed a mean average of 25.51 years (SD = 5.43). Employment was the primary source of income for 38.0% of students, while 34.7% were primarily dependent on financial support from parents, relatives or partners. In terms of living situations, about a quarter of students lived alone (26.2%), with parents or relatives (25.4%), or with a partner (25.0%). Most students were enrolled in law, economics, and social sciences (29.7%). We included students from all semesters, with the majority (41.8%) enrolled in their 7th semester or higher.

### 3.2. Bivariate Analyses

Table 2 shows the relationship between SSS, parental academic background, and students’ characteristics. The average SSS was 5.90 (SD = 1.94), with female students reporting lower values than male students (M = 5.62 vs. M = 6.17, *p* < 0.001). Students with lower SSS (M = 5.51) were more likely to reveal federal funding for education as their primary source of income, whereas those with higher SSS (M = 6.01, *p* = 0.042) primarily relied on parents, relatives or partners. Regarding living situation, students who lived with parents or relatives (M = 5.51, *p* < 0.001) had a lower average SSS than those who lived alone (M = 6.02) or with a partner (M = 6.19). Furthermore, the SSS was higher among sports students (M = 6.75, *p* = 0.004), than students in mathematics and natural sciences (M = 5.78), or the humanities (M = 5.56).

In our sample, 51.1% were first-gens, who differed by gender with a higher proportion of female first-gens (55.0% vs. 45.0%, *p* = 0.001). Financial support from family or partners was the primary source of income for 48.4% of students with two academic parents, whereas this applied to 29.4% of first-gens (*p* < 0.001). Compared to first-gens, students with two academic parents were less likely to live with their parents or relatives (14.2% vs., 28.8%, *p* < 0.001) and were more likely to attend a university instead of a university of applied sciences (75.1% vs. 65.8%, *p* = 0.028). In the field of medicine and health sciences, first-gens were underrepresented compared to students with two academic parents (6.3% vs. 14.0%, *p* = 0.037).

### 3.3. Regression Models

The linear regression models in Table 3 (see Table S1 in Supplementary Materials for an extended version) revealed that SSS was positively associated with self-rated health (β = 0.322, *p* < 0.001) and well-being (β = 0.355, *p* < 0.001). Negative associations were observed with stress (β = −0.154, *p* < 0.001), depression (β = −0.127, *p* < 0.001), and burnout (β = −0.219, *p* < 0.001). Sociodemographic, socioeconomic, and study-related models showed consistent results (Models II–IV). When stratified by gender, the association between SSS and depression was not significant for male students (β = −0.055, *p* = 0.203). Furthermore, the associations between SSS and both stress (β = −0.182 vs. β = −0.101) and burnout (β = −0.278 vs. β = −0.156) were stronger for female students.

First-gens (Ref.) indicated lower self-rated health and well-being compared to those with one (β = 0.114, *p* < 0.001; β = 0.112, *p* < 0.001) and two academic parents (β = 0.162, *p* < 0.001; β = 0.192, *p* < 0.001, Table 4 and Table S2 in Supplementary Materials). In addition, students with two academic parents had lower scores for stress (β = −0.087, *p* = 0.007) and burnout (β = −0.099, *p* = 0.002) than first-gens. These associations remained stable across all adjusted models (II–IV).

When stratified by gender, male students with two academic parents described higher self-rated health (β = 0.188 vs. β = 0.109) and well-being (β = 0.235 vs. β = 0.094) compared to female students with two academic parents. Regarding perceived stress, female first-gens (Ref.) showed higher scores compared to female students with two academic parents (β = −0.088, *p* = 0.049), while no significant difference was observed for male students (*p* = 0.174). Conversely, male first-gens (Ref.) showed differences in burnout scores compared to students with two academic parents (β = −0.119, *p* = 0.010), whereas the relationship was not significant for female students (*p* = 0.061).

### 3.4. Moderation Analyses Using Interaction Terms

Additional regression analyses included an interaction term for gender and SSS to assess whether gender moderated the associations between SSS and health outcomes (Table 5, Table S3 in Supplemental Materials). Female students reported lower levels of self-rated health (β = −0.180, *p* = 0.050) and higher levels of stress (β = 0.217, *p* = 0.025), depression (β = 0.216, *p* = 0.027), and burnout (β = 0.216, *p* = 0.023) compared to their male counterparts. Significant interactions were found between SSS and gender for depression (β = −0.219, *p* = 0.026) and burnout (β = −0.264, *p* = 0.006).

In a subsequent model that included an interaction term between gender and parental academic background, female students showed lower values for self-rated health (β = −0.122, *p* = 0.003) and well-being (β = −0.225, *p* < 0.001), and higher values for stress (β = 0.110, *p* = 0.010) compared to male students. The interaction term between having one or two academic parents and gender was significant for self-related health (β = −0.100, *p* = 0.044). This indicates that gender moderated the relationship between parental academic background and self-rated health.

## 4. Discussion

Our study highlights health inequalities among university students in Germany. Both lower SSS and first-gen status were associated with poorer self-rated health and well-being, as well as higher levels of burnout and stress, while SSS was additionally related to depression. Importantly, these associations were partly moderated by gender. This underscores the need for an intersectional perspective on understanding health inequalities in university students.

Consistent with previous research, our findings show that low SSS was associated with stress (Gellisch et al., 2024; Huynh et al., 2023; Sendatzki & Rathmann, 2022), depression (Backhaus et al., 2020; Diehl et al., 2021; Niu et al., 2023; Pössel et al., 2022), and burnout (Zahra & Hidayat, 2024). They also corroborate earlier results on the relationship between first-gen status and elevated levels of stress (Williams-York et al., 2024) and burnout (Liu et al., 2023; Williams-York et al., 2024). In contrast to previous studies (Amirkhan et al., 2023; Worthen et al., 2023), we did not observe a significant relationship between depression and first-gen status. LeBouef and Dworkin (2021) challenge the deficit-based approach to first-gen families and emphasize that first-gens often draw on particular strengths. These strengths include emotional support from their families, as well as values such as perseverance, diligence, and ambition (LeBouef & Dworkin, 2021). Such strengths may serve as protective factors against depression. Our findings on lower self-rated health and well-being among students with lower SSS and first-gen status are consistent with the smaller number of studies that have examined salutogenic indicators (Deindl et al., 2023; Niu et al., 2023; Qin et al., 2024).

A useful framework for analyzing the limited access to educational, cultural, and economic capital (Barsegyan & Maas, 2024) is provided by Bourdieu’s theory of capital (Bourdieu, 1986). In accordance with this theoretical perspective, our findings show that first-gens were significantly more dependent on funding of Federal Law on Support in Education (economic capital), more likely to live with their parents (cultural capital), and more often have chosen universities of applied sciences than universities. The choice of university type can be interpreted as a limitation in educational capital, as previous studies have shown that first-gens often lack academic role models, feel less prepared and confident in navigating the university environment, and report a lower sense of belonging within higher education institutions (Barsegyan & Maas, 2024; Drake, 2024; Gopalan & Brady, 2020; Lawrie & Kim, 2024; Rockwell & Kimel, 2025).

SSS can be understood as a psychological construct that reflects an individual’s position within the social hierarchy (Adler & Stewart, 2007; Diehl et al., 2017). SSS is closely related to personal and social identity processes. For example, low SSS among students is associated with a negative self-view and a more pessimistic perception of the world, which in turn is related to increased depressive symptoms (Pössel et al., 2022). Our findings indicate in the same way that students with lower SSS were more likely to rely on funding of Federal Law on Support in Education and to live with their parents. While these patterns resemble those observed among first-gens, they arise from a subjective rather than structural position within the social hierarchy. Taken together, this suggests that both the objective experience of social disadvantage (first-gens) and the subjective perception of social status (SSS) may contribute to social and health inequalities among students.

We identified gender differences in health outcomes. Overall, female students reported lower scores for self-rated health and well-being, as well as higher levels of stress and depression. These findings are in line with previous research, indicating that female students more frequently experienced stress (Brett et al., 2023; Gellisch et al., 2024) and depression (De Groot et al., 2024; Worthen et al., 2023), while male students tended to report higher levels of self-rated health (Kim, 2022) and well-being (Brett et al., 2023). The literature presents inconsistent findings regarding burnout by gender, with a high prevalence found in both male (Liu et al., 2023) and female students (Fiorilli et al., 2022). The mean score on the burnout dimensions of the MBI-SS in our sample was elevated (M = 3.71 on the 0–6 scale) (Gumz et al., 2013), which indicates a heightened rate of burnout. In terms of gender, we observed higher burnout prevalence among female students in connection with SSS. Conversely, higher burnout rates were found among first-gen male students, whereas the results for female first-gens were not significant.

The interactional analyses indicate that female students with lower SSS are particularly vulnerable, reporting higher levels of depression and burnout compared to male students with lower SSS. In contrast, male students appeared to benefit more from having academic parents in terms of self-rated health. This suggests that socioeconomic disadvantages may be more important for female students, while academic background may provide greater health advantages for male students. Previous research confirms that high levels of intersectionality are related to a significantly increased risk of university dropout and psychological distress (Drake, 2024). However, intersectional research within the university context remains limited (LeBouef & Dworkin, 2021). In practical terms, this suggests that support and prevention services should be designed with an intersectional perspective and implemented at multiple levels.

In line with our research question and aims, this study makes three contributions to a better understanding of health inequalities among university students:

First, by integrating both negative (stress, depression, burnout) and positive health indicators (self-rated health, well-being) within a salutogenic framework, we showed that positive health outcomes exhibited stronger associations than negative ones. This underscores the importance of incorporating an emphasis on positive health dimensions, as they appear to capture crucial aspects of students’ well-being that are often overlooked in deficit-focused approaches (Hernández-Torrano et al., 2020).

Second, by simultaneously examining SSS and first-gen status, we provide a more nuanced picture of vertical health inequalities. Although measuring health inequalities among university students is difficult (Diehl et al., 2021), we were able to show that both subjective (SSS) and objective measures (first-gen status) revealed health inequalities. Our results indicate a need for action within the German higher education system and identify how specific students’ groups can be targeted for inclusion in future interventions.

Third, by testing gender as a moderating factor, this study identified distinct intersectional patterns. For instance, low SSS was particularly detrimental for female students in terms of depression and burnout, whereas male students benefited more from parental academic background with regard to self-rated health. This extends previous research on gender differences, which described female students as a risk group (Brett et al., 2023; De Groot et al., 2024; Fiorilli et al., 2022), but has not demonstrated how gender intersects with vertical inequalities (SSS, first-gen status).

### 4.1. Recommendations for Interventions

The existing scientific literature and our results provide a basis for specific recommendations for action, which can be structured on three levels:

1. Individual level: Digital mHealth apps provide low-threshold, scalable support for stress management, particularly among students with lower SSS scores (Thomas et al., 2023). When combined with empowerment and stress management programs, these apps can further strengthen coping skills and support-reflection, potentially improving self-image and reducing depressive tendencies (Pössel et al., 2022).
2. Social level: Mentoring programs for first-gens may foster resilience and strengthen the sense of belonging (Kamalumpundi et al., 2024). Networking initiatives are also valuable, as they can help students with lower SSS and first-gens expand their connections within academic communities. In addition, burnout prevention initiatives should be implemented in the early semesters to prevent health deterioration over time (Kim, 2022; Liu et al., 2023).
3. Institutional level: Financial support for first-gens and students with lower SSS remains essential to ensure equal opportunities in health in higher education. Across all support services, it is important to ensure that students with lower SSS feel supported without experiencing additional obligation or a loss in autonomy (Xin, 2023), while also recognizing that each group, such as first-gens, brings its own strengths (LeBouef & Dworkin, 2021).

### 4.2. Limitations

The analyses revealed that health inequality is associated with a range of social determinants. By situating these associations within the broader body of international literature, we were able to classify and contextualize our findings in detail. Nevertheless, it is important to note that the results are limited to the German context and cannot be generalized to other national settings.

As all measurements are based on self-reported data, the results may be influenced by social desirability. At the same time, subjective indicators such as SSS are often more strongly correlated with health outcomes than objective measures (Haught et al., 2015; Hoebel et al., 2017), which supports the relevance of our approach.

Our sample included a slight oversampling of first-gens in Germany (51.1% compared to the national average of about 44%) (Kroher et al., 2023). Although this was unintended, we accounted for it by applying robust statistical methods, which helped to minimize potential bias while still providing valuable insight into this otherwise often underrepresented student population.

One limitation of the present study is that intelligence (IQ) was not included as a covariate. Although studies have found a correlation between the IQ of the parents and the IQ of their children (Bouchard et al., 1990; Plomin & von Stumm, 2018), first-gen status is not a mere proxy for cognitive ability. Instead, it captures a broad set of environmental dimensions, including less cultural and social capital (Barsegyan & Maas, 2024) and tend to feel a lower sense of belonging at the university (Gopalan & Brady, 2020; Lawrie & Kim, 2024). To obtain a more differential picture, future studies could incorporate cognitive factors such as IQ as well as relevant environmental outcomes including academic workload, weekly working hours for employed students, social support, and sense of belonging.

Finally, the cross-sectional design does not allow causal conclusions, although it enabled us to identify robust associations between SSS, first-gen status, and both positive and negative health outcomes within a relatively large German-wide sample, and was balanced by gender, thereby enhancing the comparability of our findings.

## 5. Conclusions

The main aim of our research was to gain a nuanced understanding of health inequalities among students by addressing both horizontal and vertical dimensions. Using an intersectional perspective, we found that both SSS and first-gen status were associated with self-rated health, well-being, burnout, and stress. In addition, lower SSS was related to higher levels of depression, underscoring the role of perceived social position as a psychosocial risk factor in the academic context. Our results further revealed gender differences and initial indications of intersectional patterns. Female students with lower SSS are particularly more affected by stress and burnout, whereas male students with academic parents benefited disproportionately more in terms of self-rated health.

These findings emphasize the need for coordinated individual, social, and institutional interventions. Future research should continue to apply an intersectional perspective within a salutogenic framework. Moreover, intervention studies should adopt a multi-level design that acknowledges the strengths of the vulnerable student groups and provides adequate resources to reduce health inequalities among university students.

## Figures and Tables

**Table 1:** Sociodemographic, socioeconomic, and study-related characteristics of the participants.

Variable	*n*	%/M (SD)
Sociodemographic characteristics		
**Gender**	1105	
Female	554	50.1
Male	551	49.9
**Age**	1089	25.51 (5.43)
**Migration background**	1098	
Yes	206	18.8
No	892	81.2
Additional socioeconomic characteristics		
**Primary source of income**	1069	
Support from parents, relatives, partner	371	34.7
Funding of Federal Law on Support in Education	183	17.1
Own employment	406	38.0
Scholarship, Loans and Others	109	10.2
**Living situation**	1088	
Alone	285	26.2
With partner	272	25.0
In shared apartment	206	18.9
Student dormitory	49	4.5
With parents or relatives	276	25.4
Study-related characteristics		
**Type of University**	1105	
University	743	67.2
University of applied sciences	362	32.8
**Area of study**	1105	
Humanities	164	14.8
Law, economics, and social sciences	328	29.7
Mathematics, natural sciences	162	14.7
Medicine, health sciences	90	8.1
Engineering	176	15.9
Art, art science	29	2.6
Sports	24	2.2
Others	132	11.9
**Semester**	1086	
1–2	197	18.1
3–6	435	40.1
>6	454	41.8

M = Mean, SD = Standard deviation, *n* = absolute frequencies, % = relative frequencies/percentages; proportion may not reach 100% due to rounding errors.

**Table 2:** Associations of SSS and first-gen status with sociodemographic, additional socioeconomic, and study-related characteristics.

	SSS			Parental Academic Background						
	1–10 (*n* = 1105, M = 5.90, SD = 1.94)			No, Neither Parent (*n* = 555, 51.1%)		Yes, One Parent (*n* = 310, 28.5%)		Yes, Both Parents (*n* = 221, 20.3%)		
Variables	*n*	M (SD)/r	*p*-Value	*n*	M (SD)/%	*n*	M (SD)/%	*n*	M (SD)/%	*p*-Value
Sociodemographic characteristics										
**Gender**	1105	5.90 (1.94)	**<0.001**	555	100.0	310	100.0	221	100.0	**0.001**
Female	554	5.62 (1.90)		305	55.0 ^x^	153	49.4	90	40.7 ^x^	
Male	551	6.17 (1.94)		250	45.0 ^x^	157	50.6	131	59.3 ^x^	
**Age**	1089	−0.057	**0.021**	552	25.67 (5.45)	304	25.13 (5.63)	220	25.70 (5.10)	0.333
**Migration background**	1098	5.91 (1.93)	0.553	553	100.0	310	100.0	221	100.0	0.170
Yes	206	5.84 (1.86)		114	20.6	51	16.5	35	15.8	
No	892	5.93 (1.95)		439	79.4	259	83.5	186	84.2	
Additional socioeconomic characteristics										
**Primary source of income**	1069	5.90 (1.93)	0.042	541	100.0	302	100.0	217	100.0	**<0.001**
Support from parents, relatives, partner	371	6.01 ^a^ (1.88)		159	29.4 ^x^	105	34.8	105	48.4 ^x^	
Funding of Federal Law on Support in Education	183	5.51 ^a^ (2.07)		105	19.4	54	17.9	23	10.6 ^x^	
Own employment	406	5.96 (1.90)		217	40.1	117	38.7	68	31.3	
Scholarship, Loans and Others	109	5.97 (1.90)		60	11.1	26	8.6	21	9.7	
**Living situation**	1088	5.90 (1.93)	**<0.001**	552	100.0	307	100.0	218	100.0	**<0.001**
Alone	285	6.02 ^a^ (1.90)		155	28.1	70	22.8	58	26.6	
With partner	272	6.19 ^b^ (1.99)		127	23.0	81	26.4	63	28.9	
In shared apartment	206	5.87 (1.92)		94	17.0	57	18.6	52	23.9	
Student dormitory	49	5.90 (2.07)		17	3.1	17	5.5	14	6.4	
With parents or relatives	276	5.51 ^ab^ (1.82)		159	28.8	82	26.7	31	14.2 ^x^	
Study-related characteristics										
**Type of University**	1105	5.90 (1.94)	0.612	555	100.0	310	100.0	221	100,0	**0.028**
University	743	5.88 (1.96)		365	65.8	203	65.5	166	75.1 ^x^	
University of applied sciences	362	5.94 (1.89)		190	34.2	107	34.5	55	24.9 ^x^	
**Area of study**	1105	5.90 (1.94)	**0.004**	555	100.0	310	100.0	221	100.0	**0.037**
Humanities	164	5.56 ^a^ (1.99)		87	15.7	48	15.5	29	13.1	
Law, economics, and social sciences	328	5.92 (1.94)		176	31.7	94	30.3	50	22.6	
Mathematics, natural sciences	162	5.78 ^b^ (1.99)		89	16.0	45	14.5	27	12.2	
Medicine, health sciences	90	6.11 (2.09)		35	6.3	23	7.4	31	14.0 ^x^	
Engineering	176	6.03 (1.98)		78	14.1	45	14.5	45	20.4	
Art, art science	29	5.34 (2.11)		17	3.1	7	2.3	5	2.3	
Sports	24	6.75 ^ab^ (1.19)		11	2.0	7	2.3	6	2.7	
Others	132	6.04 (1.64)		62	11.2	41	13.2	28	12.7	
**Semester**	1086	5.90 (1.94)	**<0.001**	549	100.0	307	100.0	219	100.0	**0.027**
1–2	197	5.76 ^a^ (1.85)		99	18.0	59	19.2	36	16.4	
3–6	435	6.23 ^ab^ (1.92)		195	35.5 ^x^	136	44.3	97	44.3	
>6	454	5.65 ^a^ (1.94)		255	46.4 ^x^	112	36.5	86	39.3	

SSS = Subjective Social Status, M = Mean, SD = Standard deviation, r = Pearson correlation coefficient, *n* = absolute frequencies, % = relative freq./percentage; proportion may not reach 100% due to rounding errors, *p*-value is based on T-tests, ANOVAs, Pearson’s correlations, and Chi^2^-tests, respectively. The indices ^a^ and ^b^ refer to significant post-hoc analyses of the ANOVAs for the variable SSS, while the index ^x^ indicates significant post-hoc analyses of the Chi^2^-tests for the variable parental academic background.

**Table 3:** Regression models of health outcomes by SSS in the total, female, and male sample.

	Model I		Model II		Model III		Model IV	
Outcome	β	*p*-Value	β	*p*-Value	β	*p*-Value	β	*p*-Value
TOTAL SAMPLE								
**Self-rated health**	**0.322**	**<0.001**	**0.291**	**<0.001**	**0.319**	**<0.001**	**0.311**	**<0.001**
**Well-being**	**0.355**	**<0.001**	**0.322**	**<0.001**	**0.346**	**<0.001**	**0.337**	**<0.001**
**Stress**	**−0.154**	**<0.001**	**−0.137**	**<0.001**	**−0.164**	**<0.001**	**−0.162**	**<0.001**
**Depression**	**−0.127**	**<0.001**	**−0.124**	**<0.001**	**−0.135**	**<0.001**	**−0.143**	**<0.001**
**Burnout**	**−0.219**	**<0.001**	**−0.255**	**<0.001**	**−0.223**	**<0.001**	**−0.234**	**<0.001**
FEMALE STUDENTS								
**Self-rated health**	**0.300**	**<0.001**	**0.296**	**<0.001**	**0.286**	**<0.001**	**0.279**	**<0.001**
**Well-being**	**0.307**	**<0.001**	**0.310**	**<0.001**	**0.311**	**<0.001**	**0.291**	**<0.001**
**Stress**	**−0.182**	**<0.001**	**−0.182**	**<0.001**	**−0.198**	**<0.001**	**−0.170**	**<0.001**
**Depression**	**−0.205**	**<0.001**	**−0.206**	**<0.001**	**−0.232**	**<0.001**	**−0.201**	**<0.001**
**Burnout**	**−0.278**	**<0.001**	**−0.285**	**<0.001**	**−0.289**	**<0.001**	**−0.263**	**<0.001**
MALE STUDENTS								
**Self-rated health**	**0.309**	**<0.001**	**0.290**	**<0.001**	**0.311**	**<0.001**	**0.304**	**<0.001**
**Well-being**	**0.355**	**<0.001**	**0.353**	**<0.001**	**0.326**	**<0.001**	**0.346**	**<0.001**
**Stress**	**−0.101**	**0.019**	**−0.094**	**0.031**	**−0.109**	**0.015**	**−0.132**	**0.003**
**Depression**	−0.055	0.203	−0.052	0.237	−0.059	0.184	−0.073	0.099
**Burnout**	**−0.156**	**<0.001**	**−0.148**	**<0.001**	**−0.151**	**<0.001**	**−0.191**	**<0.001**

SSS = Subjective Social Status, reported β are standardized. Model I: unadjusted model (without covariates); Model II: sociodemographic model (adjusted for age, migration background, gender); Model III: socioeconomic model (adjusted for primary source of income, living situation); Model IV: study-related model (adjusted for type of university, area of studies, semester).

**Table 4:** Regression models of health outcomes by first-gen status in the total, female, and male sample.

	Model I		Model II		Model III		Model IV	
Outcome	β	*p*-Value	β	*p*-Value	β	*p*-Value	β	*p*-Value
TOTAL SAMPLE								
**Self-rated health**								
No, neither parent	Ref.		Ref.		Ref.		Ref.	
Yes, one parent	**0.114**	**<0.001**	**0.101**	**0.001**	**0.114**	**<0.001**	**0.100**	**0.002**
Yes, two parents	**0.162**	**<0.001**	**0.136**	**<0.001**	**0.170**	**<0.001**	**0.158**	**<0.001**
**Well-being**								
No, neither parent	Ref.		Ref.		Ref.		Ref.	
Yes, one parent	**0.112**	**<0.001**	**0.095**	**0.002**	**0.113**	**<0.001**	**0.095**	**0.003**
Yes, two parents	**0.192**	**<0.001**	**0.159**	**<0.001**	**0.203**	**<0.001**	**0.167**	**<0.001**
**Stress**								
No, neither parent	Ref.		Ref.		Ref.		Ref.	
Yes, one parent	−0.039	0.223	−0.025	0.429	−0.031	0.341	−0.035	0.284
Yes, two parents	**−0.087**	**0.007**	**−0.069**	**0.031**	**−0.099**	**0.003**	**−0.095**	**0.004**
**Depression**								
No, neither parent	Ref.		Ref.		Ref.		Ref.	
Yes, one parent	0.013	0.691	0.016	0.631	0.019	0.568	0.008	0.813
Yes, two parents	−0.057	0.116	−0.044	0.182	**−0.074**	**0.026**	**−0.065**	**0.048**
**Burnout**								
No, neither parent	Ref.		Ref.		Ref.		Ref.	
Yes, one parent	−0.010	0.761	−0.009	0.774	0.003	0.928	−0.013	0.679
Yes, two parents	**−0.099**	**0.002**	**−0.096**	**0.003**	**−0.109**	**0.001**	**−0.104**	**0.001**
FEMALE STUDENTS								
**Self-rated health**								
No, neither parent	Ref.		Ref.		Ref.		Ref.	
Yes, one parent	0.035	0.432	0.039	0.384	0.033	0.468	0.027	0.540
Yes, two parents	**0.109**	**0.014**	**0.106**	**0.018**	**0.105**	**0.027**	**0.115**	**0.010**
**Well-being**								
No, neither parent	Ref.		Ref.		Ref.		Ref.	
Yes, one parent	0.080	0.073	0.080	0.075	0.072	0.114	0.063	0.154
Yes, two parents	**0.094**	**0.035**	**0.093**	**0.038**	**0.097**	**0.042**	0.083	0.063
**Stress**								
No, neither parent	Ref.		Ref.		Ref.		Ref.	
Yes, one parent	−0.028	0.523	−0.015	0.733	−0.020	0.655	−0.018	0.690
Yes, two parents	**−0.088**	**0.049**	−0.077	0.086	**−0.094**	**0.049**	−0.083	0.065
**Depression**								
No, neither parent	Ref.		Ref.		Ref.		Ref.	
Yes, one parent	0.031	0.483	0.038	0.401	0.042	0.360	0.033	0.463
Yes, two parents	−0.030	0.496	−0.023	0.601	−0.064	0.182	−0.031	0.488
**Burnout**								
No, neither parent	Ref.		Ref.		Ref.		Ref.	
Yes, one parent	−0.006	0.886	−0.007	0.869	0.016	0.726	−0.002	0.969
Yes, two parents	−0.083	0.061	−0.085	0.057	−0.092	0.055	−0.076	0.091
MALE STUDENTS								
**Self-rated health**								
No, neither parent	Ref.		Ref.		Ref.		Ref.	
Yes, one parent	**0.189**	**<0.001**	**0.178**	**<0.001**	**0.189**	**<0.001**	**0.179**	**<0.001**
Yes, two parents	**0.188**	**<0.001**	**0.179**	**<0.001**	**0.195**	**<0.001**	**0.183**	**<0.001**
**Well-being**								
No, neither parent	Ref.		Ref.		Ref.		Ref.	
Yes, one parent	**0.129**	**0.005**	**0.121**	**0.008**	**0.129**	**0.005**	**0.116**	**0.012**
Yes, two parents	**0.235**	**<0.001**	**0.229**	**<0.001**	**0.225**	**<0.001**	**0.206**	**<0.001**
**Stress**								
No, neither parent	Ref.		Ref.		Ref.		Ref.	
Yes, one parent	−0.038	0.411	−0.034	0.470	−0.039	0.416	−0.037	0.434
Yes, two parents	−0.063	0.174	−0.060	0.203	−0.089	0.069	−0.088	0.065
**Depression**								
No, neither parent	Ref.		Ref.		Ref.		Ref.	
Yes, one parent	−0.002	0.961	−0.004	0.936	−0.007	0.880	−0.004	0.929
Yes, two parents	−0.063	0.179	−0.056	0.235	**−0.096**	**0.049**	−0.080	0.092
**Burnout**								
No, neither parent	Ref.		Ref.		Ref.		Ref.	
Yes, one parent	−0.016	0.737	−0.012	0.789	−0.017	0.720	−0.021	0.657
Yes, two parents	**−0.119**	**0.010**	**−0.111**	**0.017**	**−0.138**	**0.004**	**−0.130**	**0.006**

SSS = Subjective Social Status, reported β are standardized. Model I: unadjusted model (without covariates); Model II: sociodemographic model (adjusted for age, migration background, gender); Model III: socioeconomic model (adjusted for primary source of income, living situation); Model IV: study-related model (adjusted for type of university, area of studies, semester).

**Table 5:** Moderation analyses on health outcomes by SSS and first-gen status using interaction terms.

Outcome	Self-Rated Health		Well-Being		Stress		Depression		Burnout	
Predictor Variable	β	*p*-Value	β	*p*-Value	β	*p*-Value	β	*p*-Value	β	*p*-Value
**SSS**										
SSS	**0.291**	**<0.001**	**0.346**	**<0.001**	**−0.101**	**0.017**	−0.059	0.170	**−0.142**	**<0.001**
Female students	**−0.180**	**<0.050**	−0.159	0.073	**0.217**	**0.025**	**0.216**	**0.027**	**0.216**	**0.023**
SSS x female students	0.034	0.711	−0.082	0.354	−0.133	0.170	**−0.219**	**0.026**	**−0.264**	**0.006**
**Parental academic background**										
One or two academic parents	**0.201**	**<0.001**	**0.196**	**<0.001**	−0.057	0.189	−0.037	0.400	−0.067	0.126
Female students	**−0.122**	**0.003**	**−0.225**	**<0.001**	**0.110**	**0.010**	0.008	0.854	−0.016	0.710
One or two academic parents x female students	**−0.100**	**0.044**	−0.083	0.086	−0.005	0.918	0.036	0.484	0.014	0.783

SSS = Subjective Social Status, reported β are standardized.

## Data Availability

The datasets generated and analyzed during the current study are not publicly available due to German data protection regulations and the assurances in the informed consent agreement and ethic approval that the data will not be disclosed, but are available from the corresponding author on reasonable request.

## Supplementary Materials

The following supporting information can be downloaded at https://www.mdpi.com/article/10.3390/ejihpe16010011/s1, Table S1: Extended version of Table 3; Table S2: Extended version of Table 4; Table S3: Extended version of Table 5. The extended Tables include the unstandardized regression coefficient (B), its standard error (SE), and the corresponding 95% confidence interval (95% CI).

## Abbreviations

The following abbreviations are used in this manuscript:

FAU	Friedrich-Alexander-Universität Erlangen-Nürnberg
First-gens	First-generation university students
M	Mean
MBI-SS	Maslach Burnout Inventory-Student Survey
PHQ	Patient Health Questionnaire
SD	Standard Deviation
SOS	Stress Overload Scale
SRH	Self-Rated Health
SSS	Subjective Social Status
WHO	World Health Organization
