# Supplementary material for: Health Inequalities in German Higher Education: A Cross-Sectional Study Reveals Poorer Health in First-Generation University Students and University Students with Lower Subjective Social Status
Source: Eur J Investig Health Psychol Educ. 2026 Jan 5;16(1):11. doi: 10.3390/ejihpe16010011 (PMC12839866; doi:10.3390/ejihpe16010011)
Supplement: Supplementary file 1 [file ejihpe-16-00011-s001.zip › ejihpe-4021415-supplementary.pdf]

Table S1. Extended version of Table 3: Regression models of health outcomes by SSS in the total, female, and male sample including B, SE, and 95% CI

	Model 1					Model 2					Model 3					Model 4				
Outcome	B	SE	95% CI	β	p-value	B	SE	95% CI	β	p-value	B	SE	95% CI	β	p-value	B	SE	95% CI	β	p-value
TOTAL SAMPLE																				
Self-rated health	0.140	0.012	0.115 – 0.164	0.322	<0.001	0.126	0.012	0.101 – 0.150	0.291	<0.001	0.138	0.013	0.113 – 0.164	0.319	<0.001	0.136	0.013	0.111 – 0.161	0.311	<0.001
Well-being	1.056	0.084	0.891 – 1.221	0.355	<0.001	0.962	0.083	0.797 – 1.125	0.322	<0.001	1.031	0.087	0.861 – 1.201	0.346	<0.001	1.007	0.085	0.840 – 1.175	0.337	<0.001
Stress	-0.084	0.016	-0.115 – -0.052	-0.154	<0.001	-0.075	0.017	-0.107 – - 0.042	-0.137	<0.001	-0.089	0.017	-0.122 – -0.056	-0.164	<0.001	-0.088	0.017	-0.121 – -0.056	-0.162	<0.001
Depression	-0.047	0.011	-0.068 – -0.025	-0.127	<0.001	-0.046	0.011	-0.068 – -0.024	-0.124	<0.001	-0.050	0.011	-0.072 – -0.028	-0.135	<0.001	-0.053	0.011	-0.075 – -0.030	-0.143	<0.001
Burnout	-0.119	0.016	-0.151 – -0.088	-0.219	<0.001	-0.123	0.016	-0.155 – -0.091	-0.255	<0.001	-0.121	0.017	-0.154 – -0.089	-0.223	<0.001	-0.128	0.016	-0.160 – -0.096	-0.234	<0.001
FEMALE STUDENTS																				
Self-rated health	0.135	0.018	0.099 – 0.171	0.300	<0.001	0.134	0.018	0.097 – 0.170	0.296	<0.001	0.129	0.019	0.091 – 0.167	0.286	<0.001	0.127	0.019	0.090 – 0.164	0.279	<0.001
Well-being	0.879	0.116	0.650 – 1.107	0.307	<0.001	0.892	0.118	0.661 – 1.123	0.310	<0.001	0.893	0.122	0.654 – 1.133	0.311	<0.001	0.841	0.118	0.610 – 1.073	0.291	<0.001
Stress	-0.100	0.023	-0.145 – -0.055	-0.182	<0.001	-0.100	0.023	-0.145 – -0.055	-0.182	<0.001	-0.109	0.024	-0.157 – -0.062	-0.198	<0.001	-0.094	0.024	-0.140 – -0.048	-0.170	<0.001
Depression	-0.071	0.015	-0.100 – -0.043	-0.205	<0.001	-0.072	0.015	-0.101 – -0.043	-0.206	<0.001	-0.081	0.015	-0.110 – -0.051	-0.232	<0.001	-0.071	0.015	-0.100 – -0.041	-0.201	<0.001
Burnout	-0.167	0.025	-0.216 – -0.119	-0.278	<0.001	-0.172	0.025	-0.221 – -0.124	-0.285	<0.001	-0.173	0.026	-0.224 – -0.123	-0.289	<0.001	-0.159	0.025	-0.209 – -0.110	-0.263	<0.001
MALE STUDENTS																				
Self-rated health	0.126	0.017	0.094 – 0.159	0.309	<0.001	0.118	0.017	0.085 – 0.151	0.290	<0.001	0.127	0.017	0.093 – 0.161	0.311	<0.001	0.124	0.017	0.090 – 0.158	0.304	<0.001
Well-being	1.031	0.116	0.802 – 1.260	0.355	<0.001	1.030	0.119	0.797 – 1.263	0.353	<0.001	0.941	0.119	0.707 – 1.174	0.326	<0.001	1.002	0.120	0.767 – 1.238	0.346	<0.001
Stress	-0.055	0.023	-0.100 – -0.009	-0.101	0.019	-0.051	0.024	-0.098 – -0.005	-0.094	0.031	-0.059	0.024	-0.106 – -0.011	-0.109	0.015	-0.071	0.024	-0.118 – -0.025	-0.132	0.003
Depression	-0.021	0.017	-0.055 – 0.012	-0.055	0.203	-0.020	0.027	-0.054 0.013	-0.052	0.237	-0.023	0.017	-0.057 – 0.011	-0.059	0.184	-0.028	0.017	-0.062 – 0.005	-0.073	0.099
Burnout	-0.078	0.021	-0.119 – -0.036	-0.156	<0.001	-0.074	0.021	-0.116 – -0.032	-0.148	<0.001	-0.075	0.022	-0.118 -0.032	-0.151	<0.001	-0.095	0.022	-0.137 – -0.052	-0.191	<0.001

SSS = Subjective Social Status, B = unstandardized regression coefficient, SE = Standard Error, CI = Confidence Interval, reported β are standardized.

Model I:	unadjusted model (without covariates)
Model II:	sociodemographic model (adjusted for age, migration background, gender)
Model III:	socioeconomic model (adjusted for primary source of income, living situation)
Model IV:	study-related model (adjusted for type of university, area of studies, semester)

Table S2. Extended version of Table 4: Regression models of health outcomes by first-gen status in the total, female, and male sample, including B, SE, and 95% CI

	Model 1					Model 2					Model 3					Model 4				
**Outcome**	**B**	**SE**	**95% CI**	**β**	**p-value**	**B**	**SE**	**95% CI**	**β**	**p-value**	**B**	**SE**	**95% CI**	**β**	**p-value**	**B**	**SE**	**95% CI**	**β**	**p-value**
TOTAL SAMPLE																				
**Self-rated health**																				
No, neither parent				Ref.					Ref.					Ref.					Ref.	
Yes, one parent	**0.212**	**0.059**	**0.097 – 0.328**	**0.114**	**<0.001**	**0.187**	**0.058**	**0.073 – 0.302**	**0.101**	**0.001**	**0.213**	**0.060**	**0.095 – 0.330**	**0.114**	**<0.001**	**0.187**	**0.059**	**0.071 – 0.304**	**0.100**	**0.002**
Yes, two parents	**0.338**	**0.066**	**0.208 – 0.467**	**0.162**	**<0.001**	**0.281**	**0.065**	**0.153 – 0.409**	**0.136**	**<0.001**	**0.351**	**0.068**	**0.218 – 0.485**	**0.170**	**<0.001**	**0.331**	**0.067**	**0.200 – 0.463**	**0.158**	**<0.001**
**Well-being**																				
No, neither parent				Ref.					Ref.					Ref.					Ref.	
Yes, one parent	**1.439**	**0.404**	**0.647 – 2.232**	**0.112**	**<0.001**	**1.225**	**0.394**	**0.452 – 1.998**	**0.095**	**0.002**	**1.449**	**0.407**	**0.650 – 2.247**	**0.113**	**<0.001**	**1.212**	**0.402**	**0.422 – 2.001**	**0.095**	**0.003**
Yes, two parents	**2.757**	**0.452**	**1.869 – 3.645**	**0.192**	**<0.001**	**2.283**	**0.441**	**1.418 – 3.147**	**0.159**	**<0.001**	**2.890**	**0.464**	**1.980 – 3.801**	**0.203**	**<0.001**	**2.392**	**0.456**	**1.498 – 3.286**	**0.167**	**<0.001**
**Stress**																				
No, neither parent				Ref.					Ref.					Ref.					Ref.	
Yes, one parent	-0.091	0.075	-0.238 – 0.056	-0.039	0.223	-0.060	0.075	-0.207 – 0.088	-0.025	0.429	-0.073	0.076	-0.222 – 0.077	-0.031	0.341	-0.081	0.075	-0.228 – 0.067	-0.035	0.284
Yes, two parents	**-0.226**	**0.084**	**-0.390 – -0.062**	**-0.087**	**0.007**	**-0.181**	**0.084**	**-0.346 – -0.016**	**-0.069**	**0.031**	**-0.256**	**0.087**	**-0.427 – -0.086**	**-0.099**	**0.003**	**-0.249**	**0.085**	**-0.416 – -0.082**	**-0.095**	**0.004**
**Depression**																				
No, neither parent				Ref.					Ref.					Ref.					Ref.	
Yes, one parent	0.020	0.051	-0.079 – 0.119	0.013	0.691	0.025	0.051	-0.076 – 0.125	0.016	0.631	0.029	0.051	-0.071 – 0.130	0.019	0.568	0.012	0.051	-0.088 – 0.112	0.008	0.813
Yes, two parents	-0.089	0.057	-0.201 – 0.022	0.057	0.116	-0.077	0.057	-0.189 – 0.036	-0.044	0.182	**-0.130**	**0.058**	**-0.245 – -0.015**	**-0.074**	**0.026**	**-0.115**	**0.058**	**-0.228 – -0.001**	**-0.065**	**0.048**
**Burnout**																				
No, neither parent				Ref.					Ref.					Ref.					Ref.	
Yes, one parent	-0.023	0.075	-0.171 – 0.125	-0.010	0.761	-0.022	0.076	-0.171 – 0.127	-0.009	0.774	0.007	0.076	-0.142 – 0.156	0.003	0.928	-0.031	0.075	-0.179 – 0.117	-0.013	0.679
Yes, two parents	**-0.259**	**0.084**	**-0.425 – -0.094**	**-0.099**	**0.002**	**-0.253**	**0.085**	**-0.419 – -0.086**	**-0.096**	**0.003**	**-0.283**	**0.087**	**-0.453 – -0.113**	**-0.109**	**0.001**	**-0.272**	**0.085**	**-0.440 – -0.105**	**-0.104**	**0.001**
FEMALE STUDENTS																				
**Self-rated health**																				
No, neither parent				Ref.					Ref.					Ref.					Ref.	
Yes, one parent	0.067	0.085	-0.100 – 0.233	0.035	0.432	0.074	0.085	-0.093 – 0.242	0.039	0.384	0.063	0.086	-0.107 – 0.232	0.033	0.468	0.052	0.084	-0.114 – 0.218	0.027	0.540
Yes, two parents	**0.252**	**0.102**	**0.051 – 0.453**	**0.109**	**0.014**	**0.243**	**0.103**	**0.042 – 0.445**	**0.106**	**0.018**	**0.241**	**0.108**	**0.028 – 0.454**	**0.105**	**0.027**	**0.267**	**0.103**	**0.065 – 0.496**	**0.115**	**0.010**
**Well-being**																				
No, neither parent				Ref.					Ref.					Ref.					Ref.	
Yes, one parent	0.970	0.540	-0.090 – 2.031	0.080	0.073	0.975	0.546	-0.098 – 2.048	0.080	0.075	0.871	0.550	-0.210 – 1.952	0.072	0.114	0.764	0.535	-0.287 – 1.815	0.063	0.154
Yes, two parents	**1.380**	**0.652**	**0.099 – 2.661**	**0.094**	**0.035**	**1.367**	**0.659**	**0.073 – 2.660**	**0.093**	**0.038**	**1.411**	**0.693**	**0.051 – 2.772**	**0.097**	**0.042**	1.217	0.654	- 0.067 – 2.502	0.083	0.063

**Stress**																				
No, neither parent				Ref.					Ref.					Ref.					Ref.	
Yes, one parent	-0.066	0.103	-0.268 – 0.136	-0.028	0.523	-0.035	0.103	-0.238 – 0.168	-0.015	0.733	-0.047	0.105	-0.254 – 0.160	-0.020	0.655	-0.041	0.103	-0.244 – 0.162	-0.018	0.690
Yes, two parents	**-0.245**	**0.124**	**-0.490 – -0.001**	**-0.088**	**0.049**	-0.214	0.125	-0.459 – 0.030	-0.077	0.086	**-0.262**	**0.133**	**-0.522 – -0.002**	**-0.094**	**0.049**	-0.233	0.126	-0.481 – 0.014	-0.083	0.065
**Depression**																				
No, neither parent				Ref.					Ref.					Ref.					Ref.	
Yes, one parent	0.046	0.066	-0.083 – 0.176	0.031	0.483	0.056	0.066	-0.075 – 0.186	0.038	0.401	0.061	0.067	-0.070 – 0.193	0.042	0.360	0.049	0.066	-0.082 – 0.179	0.033	0.463
Yes, two parents	-0.054	0.080	-0.211 – 0.102	-0.030	0.496	-0.042	0.080	-0.199 – 0.115	-0.023	0.601	-0.112	0.084	-0.278 – 0.053	-0.064	0.182	-0.056	0.081	-0.216 – 0.103	-0.031	0.488
**Burnout**																				
No, neither parent				Ref.					Ref.					Ref.					Ref.	
Yes, one parent	-0.016	0.113	-0.239 – 0.206	-0.006	0.886	-0.019	0.114	-0.243 – 0.206	-0.007	0.869	0.040	0.115	-0.185 – 0.266	0.016	0.726	-0.004	0.113	-0.226 – 0.217	-0.002	0.969
Yes, two parents	-0.257	0.137	-0.527 – 0.012	-0.083	0.061	-0.263	0.138	-0.535 – 0.008	-0.085	0.057	-0.279	0.145	-0.563 – 0.006	-0.092	0.055	-0.234	0.138	-0.506 – 0.037	-0.076	0.091
MALE STUDENTS																				
**Self-rated health**																				
No, neither parent				Ref.					Ref.					Ref.					Ref.	
Yes, one parent	**0.328**	**0.079**	**0.173 – 0.483**	**0.189**	**<0.001**	**0.308**	**0.079**	**0.152 – 0.463**	**0.178**	**<0.001**	**0.330**	**0.081**	**0.170 – 0.489**	**0.189**	**<0.001**	**0.312**	**0.080**	**0.155 – 0.470**	**0.179**	**<0.001**
Yes, two parents	**0.347**	**0.084**	**0.183 – 0.511**	**0.188**	**<0.001**	**0.325**	**0.083**	**0.162 – 0.489**	**0.179**	**<0.001**	**0.361**	**0.088**	**0.189 – 0.533**	**0.195**	**<0.001**	**0.338**	**0.086**	**0.169 – 0.507**	**0.183**	**<0.001**
**Well-being**																				
No, neither parent				Ref.					Ref.					Ref.					Ref.	
Yes, one parent	**1.598**	**0.561**	**0.496 – 2,700**	**0.129**	**0.005**	**1.508**	**0.569**	**0.391 – 2.625**	**0.121**	**0.008**	**1.592**	**0.565**	**0.483 – 2.701**	**0.129**	**0.005**	**1.436**	**0.566**	**0.323 – 2.549**	**0.116**	**0.012**
Yes, two parents	**3.079**	**0.594**	**1.912 – 4.246**	**0.235**	**<0.001**	**2.992**	**0.598**	**1.817 – 4.166**	**0.229**	**<0.001**	**2.937**	**0.609**	**1.740 – 4.134**	**0.225**	**<0.001**	**2.697**	**0.606**	**1.506 – 3.889**	**0.206**	**<0.001**
**Stress**																				
No, neither parent				Ref.					Ref.					Ref.					Ref.	
Yes, one parent	-0.089	0.108	**-0.302 – 0.124**	-0.038	0.411	-0.079	0.110	-0.295 – 0.136	-0.034	0.470	-0.090	0.111	-0.309 – 0.128	-0.039	0.416	-0.085	0.109	-0.299 – 0.128	-0.037	0.434
Yes, two parents	-0.156	0.114	**-0.380 – 0.069**	-0.063	0.174	-0.147	0.115	-0.373 – 0.079	-0.060	0.203	-0.218	0.120	-0.453 – 0.017	-0.089	0.069	-0.215	0.116	-0.444 – 0.014	-0.088	0.065
**Depression**																				
No, neither parent				Ref.					Ref.					Ref.					Ref.	
Yes, one parent	-0.004	0.078	-0.156 – 0.149	-0.002	0.961	-0.006	0.078	-0.161 – 0.148	-0.004	0.936	-0.012	0.079	-0.167 – 0.143	-0.007	0.880	-0.007	0.077	-0.159 – 0.145	-0.004	0.929
Yes, two parents	-0.111	0.082	-0.272 – 0.051	-0.063	0.179	-0.098	0.083	-0.261 – 0.064	-0.056	0.235	**-0.169**	**0.086**	**-0.337 – -0.001**	**-0.096**	**0.049**	-0.140	0.083	-0.303 – 0.023	-0.080	0.092
**Burnout**																				
No, neither parent				Ref.					Ref.					Ref.					Ref.	
Yes, one parent	-0.033	0.099	-0.228 – 0.161	-0.016	0.737	-0.027	0.100	-0.223 – 0.169	-0.012	0.789	-0.036	0.101	-0.234 – 0.162	-0.017	0.720	-0.044	0.099	-0.238 – 0.151	-0.021	0.657
Yes, two parents	**-0.269**	**0.104**	**-0.474 – -0.064**	**-0.119**	**0.010**	**0.250**	**0.104**	**-0.455 – -0.045**	**-0.111**	**0.017**	**-0.309**	**0.108**	**-0.522 – -0.096**	**-0.138**	**0.004**	**-0.292**	**0.106**	**-0.500 – -0.084**	**-0.130**	**0.006**

SSS = Subjective Social Status, B = unstandardized regression coefficient, SE = Standard Error, CI = Confidence Interval, reported β are standardized.

Model I:	unadjusted model (without covariates)
Model II:	sociodemographic model (adjusted for age, migration background, gender)
Model III:	socioeconomic model (adjusted for primary source of income, living situation)
Model IV:	study-related model (adjusted for type of university, area of studies, semester)

Table S3. Extended version of Table 5: Moderation analyses on health outcomes by SSS and first-gen status using interaction terms, including B, SE, 95% CI

		**Self-rated health**				**Well-being**				**Stress**				**Depression**				**Burnout**		
**Predictor variable**	**B**	**SE/**<br>*95% CI*	**β**	**p-value**	**B**	**SE/**<br>*95% CI*	**β**	**p-value**	**B**	**SE/**<br>*95% CI*	**β**	**p-value**	**B**	**SE/**<br>*95% CI*	**β**	**p-value**	**B**	**SE/**<br>*95% CI*	**β**	**p-value**
**SSS**																				
SSS	**0.126**	**0.017**<br>***0.092 – 0.160***	**0.291**	**<0.001**	**1.031**	**0.115**<br>***0.805 – 1.257***	**0.346**	**<0.001**	**-0.055**	**0.023**<br>***-0.100 – -0.010***	**-0.101**	**0.017**	-0.021	0.016<br>*-0.052 – 0.009*	-0.059	0.170	**-0.078**	**0.023**<br>***-0.122 – -0.033***	**-0.142**	**< 0.001**
Female students	**-0.302**	**0.154**<br>***-0.603 – -0.001***	**-0.180**	**<0.050**	**-1.832**	**1.021**<br>***-3.835 – 0.171***	**-0.159**	**0.073**	**0.455**	**0.203**<br>***0.058 – 0.852***	**0.217**	**0.025**	**0.306**	**0.138**<br>***0.035 – 0.577***	**0.216**	**0.027**	**0.457**	**0.201**<br>***0.062 – 0.852***	**0.216**	**0.023**
SSS **x** female students	0.009	0.025<br>*-0.039 – 0.058*	0.034	0.711	-0.152	0.165<br>*-0.475 – 0.170*	-0.082	0.354	-0.045	0.033<br>*-0.109 – 0.019*	-0.133	0.170	**-0.050**	**0.022**<br>***-0.093 – -0.006***	**-0.219**	**0.026**	**-0.090**	**0.032**<br>***-0.153 – 0.026***	**-0.264**	**0.006**
**Parental academic background**																				
One or two academic parents	**0.337**	**0.070**<br>***0.199 – 0.475***	**0.201**	**<0.001**	**2.272**	**0.474**<br>***1.342 – 3.202***	**0.196**	**<0.001**	-0.199	0.091<br>*-0.298 – 0.059*	-0.057	0.189	-0.052	0.062<br>*-0.174 – 0.070*	-0.037	0.400	-0.141	0.092<br>*-0.322 – 0.040*	-0.067	0.126
Female students	**-0.205**	**0.070**<br>***-0.342 – -0.069***	**-0.122**	**0.003**	**-2.597**	**0.468**<br>***-3.516 – -1.678***	**-0.225**	**<0.001**	**0.231**	**0.090**<br>***0.055 – -0.407***	**0.110**	**0.010**	0.011	0.061<br>*-0.109 – 0.131*	0.008	0.854	-0.034	0.091<br>*-0.212 – 0.144*	-0.016	0.710
One or two academic parents **x** female students	**-0.201**	**0.100**<br>***-0.396 – -0.006***	**-0.100**	**0.044**	-1.149	0.669<br>*-2.461 – 0.163*	-0.083	0.086	-0.013	0.128<br>*-0.264 – -0.238*	-0.005	0.918	0.061	0.087<br>*-0.110 – 0.232*	0.036	0.484	0.036	0.130<br>*-0.219 – 0.290*	0.014	0.783

SSS = Subjective Social Status, B = unstandardized regression coefficient, SE = Standard Error, CI = Confidence Interval, reported β are standardized.
